# Supplementary material for: Causal interplay between lactose intolerance and gut microbiota: a combined bidirectional Mendelian randomization and in vivo validation study
Source: Front Nutr. 2026 Jun 1;13:1803337. doi: 10.3389/fnut.2026.1803337 (PMC13265576; doi:10.3389/fnut.2026.1803337)
Supplement: Supplementary file 1 [file Data_Sheet_1.zip › supplementary materials/Forward/leave one out cross validation/ebi-a-GCST90027584.finngen_R12_E4_LACTONAS.pdf]

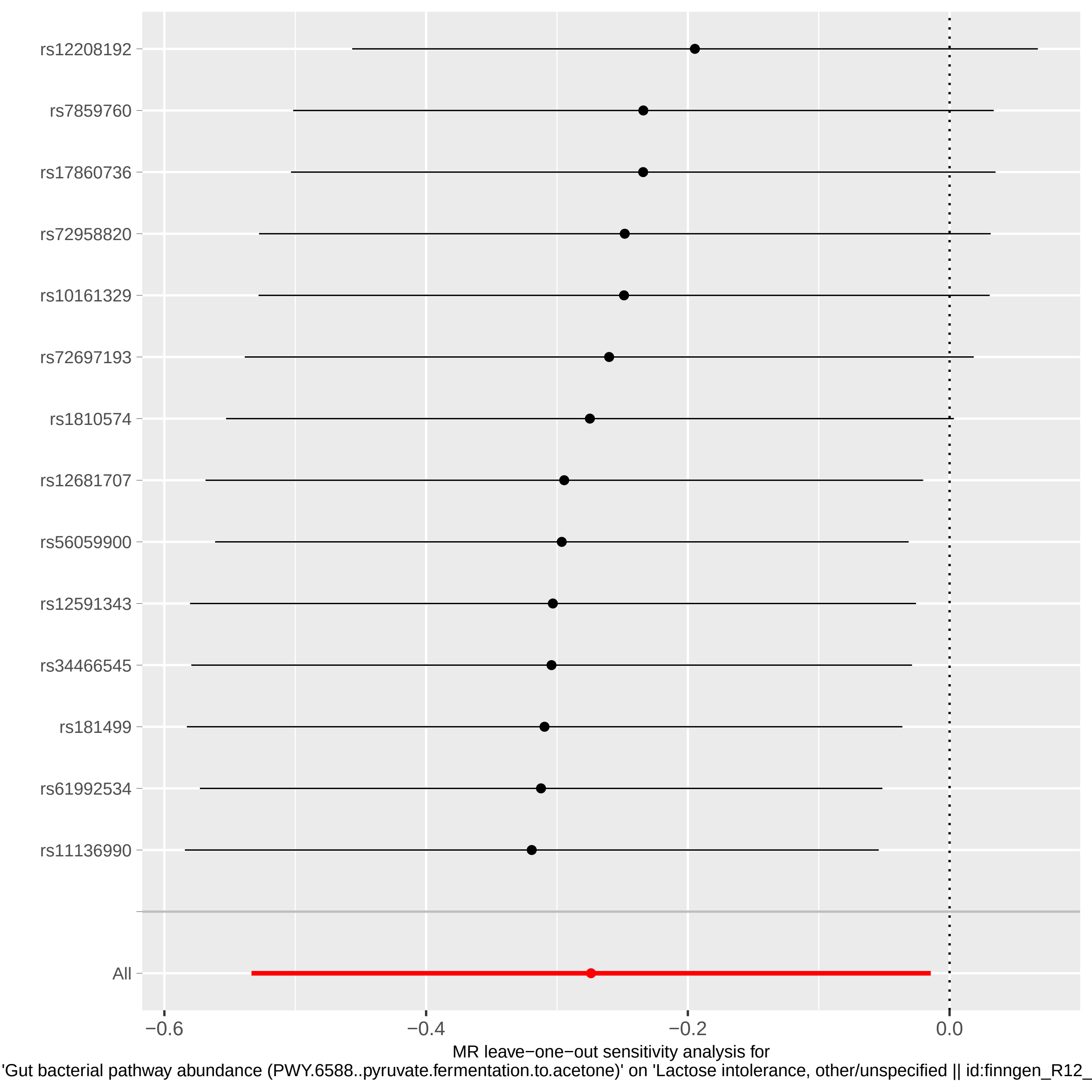
rs12208192
rs7859760
rs17860736
rs72958820
rs10161329
rs72697193
rs1810574
rs12681707
rs56059900
rs12591343
rs34466545
rs181499
rs61992534
rs11136990
All
−0.6
−0.4
−0.2
0.0
MR leave−one−out sensitivity analysis for
'Gut bacterial pathway abundance (PWY.6588..pyruvate.fermentation.to.acetone)' on 'Lactose intolerance, other/unspecified || id:finngen_R12_
